# Novel Swine Model of Respiratory Depression Induced by Fentanyl and Heroin Overdose

**DOI:** 10.21203/rs.3.rs-9228632/v1

**Published:** 2026-06-01

**Authors:** Eduardo Hatschbach, Michael D. Raleigh, Jennifer Vigliaturo, Dustin Hicks, Carly Baehr, Sandra D. Comer, Marco Pravetoni, Alonso G. P. Guedes

**Affiliations:** University of Minnesota; University of Minnesota Medical School; University of Minnesota Medical School; University of Washington School of Medicine; University of Minnesota Medical School; Columbia University Irving Medical Center; University of Washington School of Medicine; University of Minnesota

**Keywords:** Opioids, Respiratory depression, Swine model, Fentanyl, Heroin, Pharmacokinetics

## Abstract

**Background:**

This study characterized a swine model of fentanyl- and heroin-induced non-lethal apnea to support development of translational strategies for opioid overdose interventions.

**Methods:**

Spontaneously breathing, isoflurane-anesthetized Hanford swine (n = 18; 4–25 kg) received intravenous fentanyl (n = 12) or heroin (n = 6) via constant rate infusion (fentanyl: 30 μg/kg/h; heroin: 1 mg/kg/h) until production of respiratory arrest (i.e., apnea), defined as the absence of spontaneous breaths for two consecutive minutes. Fentanyl-treated pigs were either sexually immature (~ 2 months) or mature (~ 4 months); heroin-treated pigs were immature; both groups included equal numbers of males and females.

**Results:**

Results are presented as median (IQR) or mean ± SEM. Immature pigs required higher fentanyl doses to induce apnea compared with mature pigs, 17 (15–36) μg/kg vs 8 (7–10) μg/kg respectively, despite similar serum fentanyl concentration at apnea, which were 4 (3–7) and 6 (4–8) ng/mL respectively, consistent with a significantly faster clearance in immature pigs. No significant age-related differences were observed in norfentanyl concentrations or in its pharmacokinetic parameters. Heroin infusion resulted in apnea at 360 (290–502) μg/kg. Although serum heroin concentrations remained below the LLOQ throughout all time points, its metabolites 6-acetylmorphine (6-AM) and morphine serum concentration at apnea were 57 (44–91) and 34 (29–61) ng/mL, with 6-AM displaying faster clearance and a shorter half-life than morphine. Latency to resume spontaneous breathing was longer in immature than mature pigs that received fentanyl, and pigs exposed to heroin exhibited more severe respiratory depression, characterized by prolonged apnea and delayed or incomplete recovery, with a reduced response to naloxone compared with fentanyl. No statistically significant differences were detected between male and female pigs.

**Conclusion:**

In conclusion, this reproducible model enables evaluation of opioid-induced respiratory depression and its pathophysiological consequences, supporting targeted therapeutic development.

## Background:

Opioid use disorder (OUD) and opioid-related fatalities have reached epidemic levels, driven largely by the proliferation of potent synthetic opioids such as fentanyl [1, 2]. Opioid-induced respiratory depression (OIRD), marked by reduced respiratory rate and tidal volume, is the leading cause of death in opioid overdoses and can result in fatal hypoxia if not promptly treated [3, 4]. While heroin remains a concern, fentanyl’s potency, rapid onset [5], and narrow margin of safety [6], make it particularly lethal, a risk magnified by its growing presence in the illicit drug supply and the unpredictability of street formulations [7].

Despite progress in opioid research, critical gaps remain in understanding how factors like sex, age, and genetics influence opioid effects and overdose risk [8, 9]. Evidence suggests sex-based differences in susceptibility to OIRD, likely due to hormonal, metabolic, and receptor-level variations [10, 11, 12]. Alarming increases in fentanyl-related deaths have also emerged among youth, with mortality rising more than 30-fold in children and adolescents from 2013 to 2021, and even further from 2018 onward [13, 14, 15]. Heroin has also contributed to overdose deaths in this population [16]. Given that age-related physiological differences can impact drug response [17, 18], there is a need for animal models that can better capture opioid effects across age and sex.

Naloxone remains the first-line treatment for overdose, but its limitations, including delayed onset and the need for repeated dosing, highlight the need for improved strategies [19, 10, 20, 21]. Novel therapies such as opioid-targeting vaccines and monoclonal antibodies show promise for long-term protection against synthetic opioids [22, 23, 24].

Large animal models like miniature swine offer translational relevance due to their physiological similarities to humans, especially in respiratory and cardiovascular systems [25, 26, 27]. In this study, we developed and characterized a pig model of non-lethal OIRD produced by fentanyl and heroin, including determination of serum drug/metabolite levels, basic pharmacokinetic analyses, and recovery. By including sex and age as biological variables, we aim to deepen the understanding of opioid-related pharmacokinetic and pharmacodynamic (PK/PD) effects and support the development of targeted interventions for OIRD.

## Methods

### Animals

Eighteen Hanford miniature swine (Sinclair BioResources, Auxvasse, MO) of two age groups were used in the study, representing sexually immature (~ 2 months old) and mature (~ 4 months old) animals. Twelve pigs (n = 6 males and n = 6 females) were challenged with fentanyl (n = 5 immature and n = 7 mature) and 6 pigs were challenged with heroin (n = 3 males and n = 3 females; all immature) under light isoflurane anesthesia. After data collection and with the pigs still under isoflurane anesthesia, euthanasia was accomplished with an overdose of pentobarbital sodium (> 90 mg/kg) via rapid intravenous injection. Death was confirmed by the absence of arterial and ECG waveforms, audible heart sounds, and corneal reflex. The study protocol was reviewed and approved by the University of Minnesota Institutional Animal Care and Use Committee.

### Anesthesia and instrumentation

Pigs were anesthetized with isoflurane (Isospire, Dechra Pharmaceuticals, Northwich, UK) delivered using an agent-specific, calibrated vaporizer in 100% oxygen. Isoflurane was administered initially via facemask for induction of anesthesia, and then via a cuffed, Murphy-type orotracheal tube for maintenance of a light plane of anesthesia sufficient to prevent spontaneous movement. This procedure allowed the pigs to breathe spontaneously while positioned in lateral recumbency. Auricular veins were aseptically cannulated bilaterally with 22- or 24-gauge, 1.0-1.5-inch-long over-the-needle catheters for fluid (lactated Ringer’s solution, 5 mL/kg/h) and opioid administration. To facilitate arterial blood pressure monitoring and blood sampling, the saphenous artery was aseptically cannulated using a 22-gauge, 1.5-inch-long over-the-needle catheter via a small cut-down procedure, and the catheter was secured in place with 2 – 0 monocryl suture. Additional instrumentation included lead-II electrocardiogram, core body temperature via an esophageal temperature probe, sidestream capnography and spirometry using a multiparameter monitor Datex-Ohmeda Compact S5 (GE Healthcare, Chicago, IL, USA), and transmittance pulse oximetry probe positioned on the ear (PalmSAT 2500 Series, Nonin Medical Inc., Plymouth, MN, USA).

### Opioid-induced critical respiratory depression protocol

Fentanyl (30 μg/kg/h) or heroin (1 mg/kg/h) were administered as continuous intravenous infusions until production of respiratory arrest (i.e., apnea). For the purposes of this study, apnea was defined as the absence of spontaneous breaths for two consecutive minutes. If the apnea endpoint was not achieved within an hour, the opioid infusion rate was doubled each subsequent hour, up to a maximum of 3 hours. The total dose required to produce apnea was recorded. The opioid infusion was discontinued once apnea was confirmed, and the latency to return to regular spontaneous breathing was recorded. Regular spontaneous breathing was defined as the resumption of spontaneous breaths without relapsing into apnea as defined above.

### Serum opioid quantification

Serum concentrations of fentanyl, norfentanyl, heroin, 6-acetylmorphine (6-AM), and morphine were measured at predefined timepoints, including the onset of apnea, and when pigs resumed spontaneous breathing after discontinuing opioid infusion. Serum concentrations of both opioids and their metabolites were additionally determined at 1, 2, 4, 8, 16, 32, and 60 minutes after discontinuation of drug administration for calculation of basic pharmacokinetic parameters (see below). Opioid/metabolite concentrations were determined by liquid chromatography/mass spectrometry (LC-MS) as in our previous studies [23, 28]. The assay’s lower limit of quantification (LLOQ) was 0.5 ng/ml for fentanyl and norfentanyl, and 1 ng/ml for heroin, 6-AM, and morphine.

### Pharmacokinetic calculations

Pharmacokinetic parameters for fentanyl, heroin, and their metabolites were derived using non-compartmental analysis (NCA). Plasma concentration–time data obtained after cessation of the infusion were used to calculate the area under the concentration–time curve (AUC) using the linear trapezoidal method. Clearance (Cl) was calculated as the total administered dose divided by AUC (Cl = Dose/AUC). The apparent volume of distribution (Vd) was estimated from the ratio of dose to plasma concentration at the first post-infusion sampling point (Vd = Dose/C_0_), where C_0_ represents the serum concentration at two minutes of apnea (M2). The terminal half-life (t½) was calculated using the standard relationship as t½ = 0.693*Vd/Cl.

### Respiratory and cardiovascular parameters

Respiratory rate (*f*), end-tidal carbon dioxide tension (EtCO_2_), tidal volume (TV), minute volume (MV), and peripheral oxygen saturation (SpO_2_) were recorded every minute throughout the experiment. Arterial tensions of oxygen (PaO_2_) and carbon dioxide (PaCO_2_), and arterial hemoglobin oxygen saturation (SaO_2_) were obtained before starting opioid infusion (baseline), and at specific respiratory events during and after opioid infusion including at the onset of apnea, at the return to spontaneous breathing, and at 32 and 60 minutes after the opioid infusion was discontinued. Arterial blood samples for gas analysis were collected in commercial blood gas syringes (BD Arterial Blood Gas Syringes, Becton, Dickinson and Company, Franklin Lakes, NJ, USA), kept in ice under anaerobic conditions and analyzed within 30–60 minutes of collection using a clinical grade, calibrated portable blood gas analyzer (I-STAT CG4 test cartridges; Abbott Point Care Inc, NJ, USA). The resulting impact on cardiovascular function was also of interest given its importance in respiratory gas exchange and delivery. Thus, heart rate (HR) and rhythm and mean arterial pressure (MAP) were monitored throughout the experiment and recorded at the same time points as for arterial blood gas tensions described above.

### Opioid reversal with naloxone

At 60 minutes post-infusion, in cases where *f* and/or EtCO_2_ had not returned to pre-opioid baseline (M0), naloxone was administered intravenously at bolus doses of 0.25 mg/kg every two minutes for a maximum of three doses (maximum cumulative dose of 0.75 mg/kg) or until *f* and/or EtCO_2_ returned to the pre-opioid infusion baseline, whichever came first.

### Statistics

Statistical analyses were performed using GraphPad Prism Version 10.3.1 for MAC OS (GraphPad Software, Inc., CA, USA). Normality was assessed by visual inspection of QQ plots and the Shapiro-Wilk normality test. Accordingly, data were analyzed using two-tailed Mann-Whitney tests or mixed-effects model followed by Holm-Šídák’s multiple comparisons test while correcting for multiple comparisons using statistical hypothesis testing. Statistical significance was set at p < 0.05. Unless otherwise noted, data are presented as median and interquartile range (IQR) or mean ± SEM.

## Results

### Animals and Anesthesia

The median (ranges) ages of the pigs were 2.5 (2-3.1) months for the sexually immature animals and 4.1 (3.9-5.9) months for the sexually mature animals, and their respective median (range) body weights were 6.7 kg (3.8-9.9) and 14.5 kg (13.7-22). Induction of anesthesia with 4-5% isoflurane delivered in 100% oxygen via a tight-fitting face mask allowed for a calm, smooth, and well-controlled transition to general anesthesia without observable agitation or adverse reactions in all pigs. Orotracheal intubation with an appropriately sized cuffed tracheal tube was easily performed without complications or difficulties. A light plane of anesthesia was maintained with end-tidal isoflurane concentrations between 1.9-2.2% in all animals.

### Opioid dose to induce apnea

**Figure 1:**
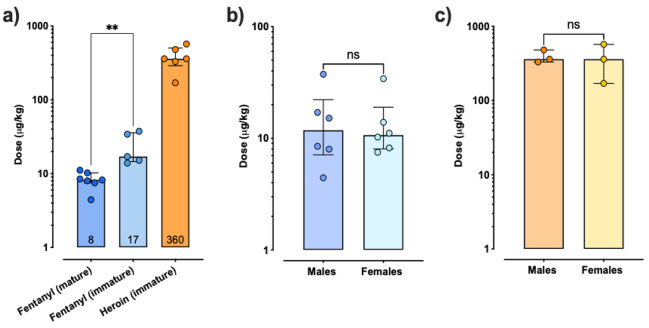
Fentanyl induces apnea more potently than heroin and exhibits age-related differences in potency. Doses of fentanyl and heroin associated with apnea, aggregated and separated by sex and age (fentanyl only), were determined using fentanyl or heroin infusions in Hanford minipigs lightly anesthetized with isoflurane. a) Doses of fentanyl and heroin associated with apnea with both sexes combined in sexually immature and mature pigs; b) Doses of fentanyl associated with apnea in male and female pigs. c) Dose of heroin associated with apnea in male and female pigs. Data are median (also shown numerically within the bars), interquartile range (IQR) and individual values (ns = not significant; * p < 0.05, ** p < 0.01; two-tailed Mann-Whitney test).

The doses of fentanyl and heroin required to induce apnea are shown in Figure 1. There were no significant sex-related differences in the doses of fentanyl and heroin required to cause apnea in these pigs. However, the dose of fentanyl to induce apnea was significantly higher in immature than mature pigs (median 17μg/kg , IQR 14-36 vs. median 8 μg/kg, IQR 7-10, respectively). Given the infusion rates selected, the latency to apnea mirrored the dose-response findings as shown in the **Additional file Figure A1.**

### Serum opioid concentrations at apnea and return to spontaneous breathing

**Figure 2:**
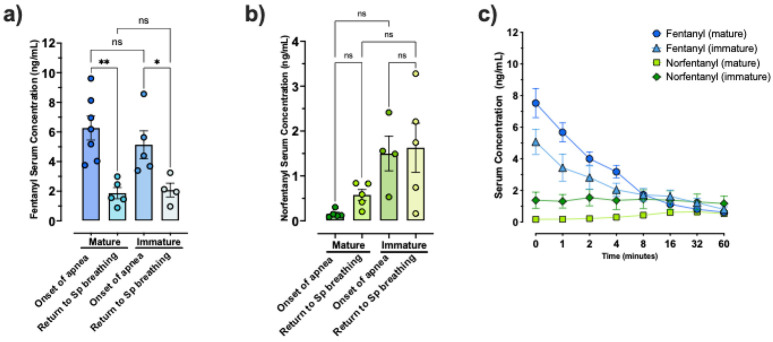
Serum concentrations of fentanyl and norfentanyl associated with apnea and return to spontaneous breathing. Serum concentrations of fentanyl and norfentanyl were determined in immature (2 months old) and mature (4 months old) Hanford minipigs lightly anesthetized with isoflurane. Concentrations were determined at several endpoints of interest, including at onset of apnea, at return to spontaneous breathing after stopping the infusion and at 1, 2, 4, 8, 16, 32 and 60 minutes after stopping the infusion. a) Serum concentration of fentanyl at onset of apnea and at return to spontaneous breathing. b) Serum concentration of norfentanyl at onset of apnea and at return to spontaneous breathing. c) Serum concentration of fentanyl and norfentanyl over time after stopping fentanyl infusion. Data are mean±SEM and individual values (ns = not significant; * p < 0.05, ** p < 0.01; mixed-effects model followed by Holm-Šídák’s multiple comparisons test while correcting for multiple comparisons using statistical hypothesis testing).

Serum concentrations of fentanyl, heroin, and their metabolites were determined at the onset of apnea (OA) and at the return of spontaneous breathing (RSB) after stopping the infusion. For fentanyl, median serum concentrations were not significantly different between immature and mature pigs at OA (4.4 ng/mL, IQR 3.5-7.1 and 6.2 ng/mL, IQR 4.0-8.1, respectively) or at RSB (2.0 ng/mL, IQR 1.2-3.0 and 1.5 ng/mL, IQR 1.2-2.7, respectively), but concentrations at OA (4.4 and 6.2 ng/mL) were significantly higher than at RSB (2.0 and 1.5 ng/mL) for both age groups. No significant differences were found between males and females at OA (6.2 ng/mL, IQR 4.8–9.0 vs. 4.0 ng/mL, IQR 3.7–7.0) or RSB (2.0 ng/mL, IQR 1.5–3.2 vs. 1.8 ng/mL, IQR 1.0–2.6). For norfentanyl, serum concentrations were numerically higher in immature compared to mature pigs at both OA (1.5 ng/mL, IQR 0.8–2.2 vs. 0.12 ng/mL, IQR 0.1–0.2) and RSB (1.7 ng/mL, IQR 0.4–2.7 vs. 0.6 ng/mL, IQR 0.3–0.8), but these differences were not statistically significant. Sex-related differences were not observed at OA (1.5 ng/mL, IQR 0.1–2.4 in males; 0.2 ng/mL, IQR 0.2–0.8 in females) or RSB (2.2 ng/mL, IQR 0.6–3.2 in males; 0.7 ng/mL, IQR 0.2–0.8 in females), nor between these endpoints. Additional time point concentrations are presented in Figure 2.

**Figure 3:**
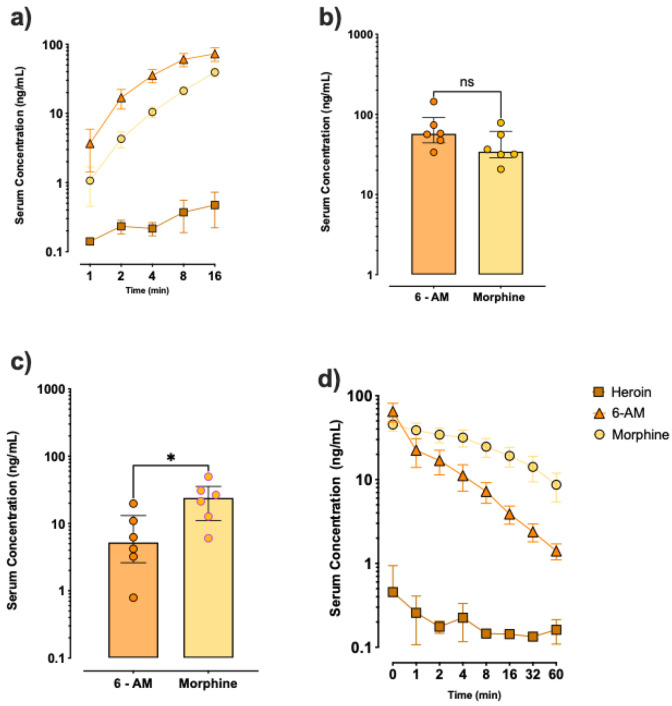
Heroin is rapidly converted to 6-acetylmorphine (6-AM) and morphine in pigs. a) Serum concentration of heroin and its metabolites (6-AM and morphine) during heroin infusions in Hanford minipigs lightly anesthetized with isoflurane. b) Serum concentration of 6-AM and morphine at onset of apnea. c) Serum concentration of 6-AM and morphine at return to spontaneous breathing after stopping the infusion. d) Serum concentration of heroin, 6-AM and morphine over time after stopping heroin infusion. Data are Mean±SEM (a, d) or median, interquartile range (IQR) and individual values (b, c; ns = not significant; * p < 0.05; two-tailed Mann-Whitney test). Note that serum heroin concentrations (panels a, d) were below the lower limit of quantification (LLOQ = 1 ng/mL) at all time points, values are shown for completeness but are not considered quantitatively reliable.

Serum heroin concentrations remained below the LLOQ throughout all time points. Serum concentrations of its main metabolites, morphine, and 6-AM, are shown in Figure 3. At the onset of apnea (OA), the concentration of 6-AM (median 57 ng/mL; IQR 44-91) was 1.7-fold higher than morphine (median 34 ng/mL; IQR 29-61). This relationship reversed when pigs returned to spontaneous breathing (RSB), such that the median serum concentration of 6-AM (5 ng/mL; IQR 3-13) was nearly 5-fold lower than that of morphine (24 ng/mL; IQR 11-35). Serum concentrations of both 6-AM and morphine were lower at RSB relative to OA, although the reduction was comparatively greater for 6-AM (decreased from 57 to 5 ng/mL, 91% reduction) than morphine (decreased from 34 to 24 ng/mL; 29% reduction). There were no sex-related differences within or between those endpoints. Based on serum concentrations at the onset of apnea, fentanyl was approximately 6-fold more potent than morphine and 10-fold more potent than 6-AM in causing apnea in our pig model.

### Pharmacokinetic parameters

**Figure 4:**
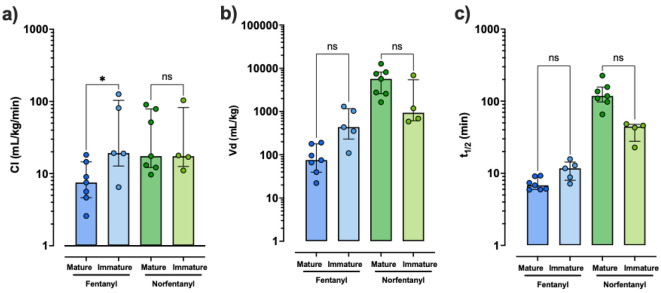
Sexual maturity significantly influences fentanyl clearance in pigs. Pharmacokinetic parameters of fentanyl and norfentanyl in sexually immature and mature pigs following cessation of the infusion at two minutes into apnea. a) Clearance (Cl). b) Volume of distribution (Vd). c) Terminal elimination half-life (t½). Data are median and interquartile range (IQR) and individual values (ns = not significant; * p < 0.05; Kruskal-Wallis test with Dunn’s multiple comparisons test while correcting for multiple comparisons using statistical hypothesis testing).

Pharmacokinetic parameters for fentanyl and norfentanyl were calculated based on serum concentrations following cessation of the infusion and are shown in Figure 4 and **Additional file Table A1**. For fentanyl, clearance (Cl) was significantly slower in mature (median 7 mL/kg/min, IQR 5-14) than immature (median 19 mL/kg/min, IQR 13-103) pigs. Although the volume of distribution (Vd) for fentanyl was lower in mature pigs, this difference was not statistically significant. However, immature pigs had a longer terminal elimination half-life (t½) compared to mature animals, but these differences did not reach statistical significance. For norfentanyl, the pharmacokinetic parameters did not differ significantly between age groups. However, mature pigs showed a trend toward higher Vd and longer t½ compared to immature pigs.

**Figure 5:**
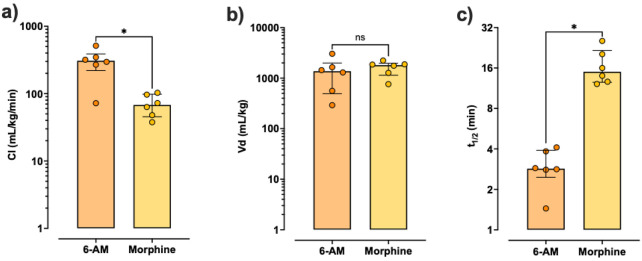
Of heroin metabolites, 6-acetylmorphine (6-AM) displays faster clearance and shorter t/12 than morphine in pigs. Pharmacokinetic parameters of heroin metabolites 6-AM and morphine in sexually immature pigs (~2 months old) following cessation of the infusion at two minutes into apnea. a) Clearance (Cl); b) Volume of distribution (Vd); c) Terminal elimination half-life (t½). Data are median and interquartile range (IQR) and individual values (ns = not significant; * p < 0.05, two-tailed Wilcoxon matched-pairs signed rank test).

The pharmacokinetic disposition of the two major heroin metabolites, 6-AM and morphine, was evaluated in immature pigs. Serum heroin concentration remained below the LLOQ at all sampled time points and thus pharmacokinetics parameters were not calculated. The Cl was significantly higher for 6-AM compared to morphine while the Vd did not differ between these two metabolites. As expected, t½ was significantly longer for morphine compared to 6-AM. These results are illustrated in Figure 5 and in **Additional file Table A1**.

### Return to spontaneous breathing after apnea

**Figure 6:**
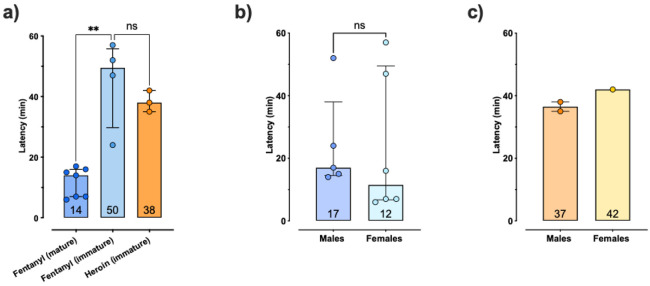
Latency to return to spontaneous breathing after apnea induced by fentanyl was age-dependent. Latency to return to spontaneous breathing after apnea induced by fentanyl and heroin infusions aggregated and separated by sex and sexual maturity (fentanyl only) in Hanford minipigs lightly anesthetized with isoflurane. a) Latency to return to spontaneous breathing after discontinuing fentanyl infusion in sexually immature (2 months old) and mature (4 months old) and heroin infusion with both sexes combined (note: n=1 animal challenged with fentanyl and n=3 animals challenged with heroin did not resume spontaneous breathing during the 60-minute observation period); b) Latency to return to spontaneous breathing after discontinuing fentanyl infusion in male and female pigs; c) Latency to return to spontaneous breathing after discontinuing heroin infusion in male and female pigs; Data are median, interquartile range (IQR) and individual values (ns = not significant; ** p < 0.01; a: Kruskal-Wallis test with Dunn’s multiple comparison test corrected for multiple comparisons using statistical hypothesis testing; b: unpaired two-tailed Mann-Whitney test).

Once apnea was confirmed, the opioid infusion was discontinued and the latency to resume spontaneous breathing was recorded over a 60-minute observation period. Three immature animals from the heroin group (one male and two females) and a single immature male from the fentanyl group did not regain spontaneous breathing. For fentanyl, the median latencies to return to spontaneous breathing were 17 minutes in males (IQR 15-38) and 12 minutes in females (IQR 7-50). This difference was not statistically significant. In contrast, age (i.e., sexual maturity) had a significant effect on latency to return to spontaneous breathing, with immature animals taking longer (median 50 minutes; IQR 30-56) than mature animals (median 14 minutes; IQR 7-16). For heroin, the median latency to return to spontaneous breathing was 37 minutes in males (IQR 35-38) and 42 minutes in the single female who resumed spontaneous breathing during the evaluation period. Pigs challenged with fentanyl recovered respiratory function markedly better than those challenged with heroin, as indicated by superior minute ventilation and arterial CO2 tensions, and as illustrated in Figure 6 and **Additional file Table A2**.

### Additional respiratory, cardiovascular and metabolic parameters

**Figure 7:**
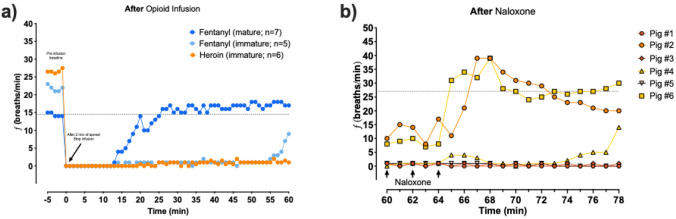
Fentanyl shows age-dependent respiratory depressant effects in pigs. Respiratory rate (f) was recorded every minute in immature (2 months old; fentanyl and heroin) and mature (4 months old; fentanyl only) Hanford minipigs lightly anesthetized with isoflurane and challenged with fentanyl and heroin infusions. a) Respiratory rate after fentanyl and heroin infusion. b) Respiratory rate of each pig in the heroin group after naloxone administration. Only the median respiratory rates are shown to improve clarity.

**Additional file Table A2** shows several additional respiratory parameters (respiratory volumes and gas tensions) determined before, during, and after fentanyl or heroin infusions. Respiratory rates minute-by-minute throughout the experiment for both opioids are shown in Figure 7. Pre-opioid baseline respiratory parameters under a light plane of isoflurane anesthesia are presented in **Additional file Table A3**. Heart rate, mean arterial pressure, arterial blood gas and limited metabolic values before, during, and after fentanyl and heroin exposure are shown in **Additional file Table A4**.

### Naloxone

Intravenous naloxone was administered at 0.25 mg/kg increments every 2 minutes up to a total cumulative dose of 0.75 mg/kg to pigs whose respiratory rate and/or EtCO2 had not returned to pre-opioid baseline within 60 minutes post-infusion. Accordingly, naloxone was required in only 1 animal from the fentanyl group, an immature male pig (2 months old), and this pig never recovered spontaneous breathing despite having received the full cumulative naloxone dose of 0.75 mg/kg. All 6 animals in the heroin group required naloxone administration after the conclusion of data collection, but restoration of respiratory function was effective in only 2/6 pigs. In the remaining four animals (one male and three females), even the maximum naloxone dose of 0.75 mg/kg failed to restore spontaneous breathing. Among the two animals that responded to naloxone, both were male and recovered following the initial 0.25 mg/kg dose. Notably, naloxone was effective in successfully restoring the respiratory rate to pre-opioid infusion baseline only in pigs that were showing some evidence of spontaneous breaths. In contrast, none of the four pigs failing to respond to naloxone had signs of spontaneous breathing activity.

## Discussion

This study aimed to develop and characterize a non-lethal swine model of critical respiratory depression induced by opioid overdose as a large animal platform for investigating opioid pharmacology and for developing strategies aimed at mitigating the impact of the opioid epidemic. Critical respiratory depression characterized by respiratory arrest was reliably induced with fentanyl and heroin. For the purposes of this model, apnea was defined as absence of spontaneous breaths for 2 minutes, to provide a useful endpoint for the development of strategies to counteract the impact of opioid overdoses (23, 28). For example, this clear and robust endpoint has facilitated testing the efficacy of an anti-fentanyl vaccine, where the fentanyl dose to cause apnea was significantly higher in the immunized pigs compared to vehicle controls (23).

Another unique feature of this model is that animals remain alive during the prolonged apnea. This is possible due to the high arterial oxygen tension created by high fraction of inspired oxygen throughout anesthesia, combined with apneic oxygenation during respiratory arrest (29) and decreased metabolic needs during anesthesia. By discontinuing the opioid infusions at the apnea endpoint and allowing the pigs to recover spontaneously, we were able to determine what the latency would be for an individual to recover from an opioid overdose leading to respiratory arrest, including the impact of sexual maturity in the case of a fentanyl overdose. Our results revealed that the respiratory depressant effects of fentanyl lasted significantly longer in sexually immature (median 50 minutes) than in mature (median 14 minutes) animals regardless of sex. In sexually immature animals, latency to return to spontaneous breaths in those animals who resumed spontaneous breathing was not significantly different between fentanyl and heroin. However, the respiratory effects of heroin were markedly more profound than those of fentanyl given that only 3/6 animals overdosed with heroin resumed spontaneous breathing compared to 4/5 animals overdosed with fentanyl. Thus, this model is suitable for testing novel strategies to reverse the respiratory effects of opioids, such as monoclonal antibodies (23), as well as for longitudinal appraisal of a scenario of critical respiratory depression caused by an overdose of fentanyl or heroin, including the role played by sex and sexual maturity among other biologically relevant variables.

We also compared aspects of pharmacokinetics/pharmacodynamics of fentanyl in sexually immature and mature animals because sexual maturity is known to influence drug pharmacokinetics [30, 31, 32] and fatal fentanyl overdoses have increased faster in adolescents than adults in recent years [13, 14, 15, 16]. Our finding of significantly higher serum fentanyl clearance in sexually immature than in mature pigs is consistent with age-related differences reported in humans, which are thought to result from proportionally higher liver blood flow in immature individuals (33). This is also likely present in pigs given their similar hepatic blood supply as humans (34). Further, immature pigs had markedly higher heart rates and mean arterial pressures than mature pigs, suggesting that their cardiac output, and consequently hepatic blood flow, was also higher. Despite significantly faster fentanyl clearance, immature pigs had significantly longer latency to return to spontaneous breathing after an overdose compared to mature animals. Thus, the observed difference in respiratory depressant effects cannot be explained by the pharmacokinetic disposition of fentanyl, indicating that other factors may be at play. For example, the longer latency to return to spontaneous breathing might have resulted from higher norfentanyl serum concentrations in immature animals, even though the difference was not statistically significant (likely a type II error). Our results reinforced the need for age-specific considerations in both opioid dosing and reversal strategies, and our pig model can serve as a platform to explore biologically relevant variables and advance the understanding of opioid pharmacology.

Serum heroin concentrations were below LLOQ (1 ng/mL) throughout the entire experiment, consistent with the rapid in vivo degradation of heroin to 6-AM and subsequently morphine [35, 36]. Notably, heroin may continue to degrade to 6-AM and morphine after blood collection unless samples are immediately stabilized [36]. Serum 6-AM concentrations were nearly twice as high as that of morphine at the onset of apnea, and nearly half that of morphine at return to spontaneous breathing. Additionally, return to spontaneous breathing coincided with a 91% reduction in serum concentrations for 6AM compared to only 29% reduction for morphine. This is supported by the pharmacokinetic analysis demonstrated that 6-AM displayed a markedly faster serum clearance and shorter half-life compared with morphine. Thus, these results suggest that 6-AM might represent the main heroin metabolite associated with respiratory depression in this pig model, corroborating prior results in rats that were directly injected with two different doses of heroin, 6-AM or morphine [37]. On the other hand, a previous pharmacokinetic/pharmacodynamic study in humans administered intravenous and intranasal heroin suggested that morphine, and not 6-AM, is more closely associated with the pharmacodynamic effects of heroin [38].

Our results also revealed that fentanyl was 33-fold more potent than heroin in producing respiratory depression in our pig model, consistent with the 10-fold difference reported in rats [39] and with the known higher potency of fentanyl compared to morphine in humans. However, the respiratory depressant effects of heroin were more marked than those of fentanyl. Naloxone was administered to all six pigs overdosed with heroin and appeared effective in only two males. In contrast, three female pigs and one male did not respond, even at the highest naloxone dose tested. A systematic review suggests gonadal hormones may affect opioid responses, though findings in different studies were somewhat inconsistent [40]. Clinical observations indicate older age and male sex as risk factors for opioid-induced respiratory depression in humans [41]. Previous studies have reported sex-based differences in opioid metabolism and μ-opioid receptor sensitivity, potentially influenced by hormonal factors [42, 43]. Clinical observations similarly suggest that women may require higher naloxone doses due to differences in metabolism and receptor distribution [43, 44]. Consistent with these observations, these findings suggest the swine model may be useful for investigating sex-specific mechanisms of opioid-induced respiratory depression and for informing more personalized naloxone reversal strategies.

Limitations of the present study include insufficient statistical power to detect subtle differences in drug response across sex and treatment conditions, although proof-of-concept evidence is provided that this is a suitable platform to explore these questions in specifically designed studies. While two different age ranges were used for fentanyl due to evidence of increased fentanyl overdose lethality in adolescents [13, 14, 15, 16], only sexually immature animals were used to study heroin, such that no age-related information can be derived for this opioid. The provision of 100% inspired oxygen prevented us from studying opioid-induced hypoxia and lethality, although it enabled the study of apnea as a robust endpoint of non-lethal critical respiratory depression and determination of return to spontaneous breathing, along with measuring serum drug concentrations, that would not have been possible if the pigs were breathing room air (21% inspired oxygen). Importantly, all data were collected under a background of isoflurane anesthesia, which does not reflect the clinical scenario of human opioid overdose, where anesthesia is absent.

## Conclusion

We report the successful development and characterization of a preclinical model of non-lethal critical respiratory depression characterized by apnea induced with fentanyl or heroin in Hanford miniature swine. Our findings provide insights into the pharmacokinetics and pharmacodynamics of these opioids, laying the foundation for improving therapeutic strategies aimed at mitigating OIRD and enhancing clinical outcomes in opioid overdose treatment.

## Supplementary Material

This is a list of supplementary files associated with this preprint. Click to download.

• AdditionalfilesNovelSwineModelofRespiratoryDepressionInducedbyFentanylandHeroinOverdoseFINALVERSION.docx

## Data Availability

All data generated or analyzed during this study are included in this published article and its supplementary information files. Data files are available upon request to the corresponding author.

## Abbreviations

OUD: Opioid use disorder
OIRD: Opioid-induced respiratory depression
PK: Pharmacokinetic
PD: Pharmacodynamic
6-AM: 6-acetylmorphine
OA: Onset of apnea
RSB: Return to spontaneous breathing
LC-MS: Liquid chromatography/mass spectrometry
LLOQ: Lower limit of quantification
AUC: Calculate the area under the concentration–time curve
Cl: Clearance
Vd: Volume of distribution
C_0_: Extrapolated initial concentration
t½: Terminal half-life
*f*: Respiratory rate
EtCO_2_: End-tidal carbon dioxide tension
TV: Tidal volume
MV: Minute volume
SpO_2_: Pulse oximetry
PaO_2_: Arterial tensions of oxygen
PaCO_2_: Arterial tensions of carbon dioxide
SaO_2_: Arterial oxygen saturation
HR: Heart rate
MAP: Mean arterial pressure
IQR: Interquartile range
ns: Not significant
ADME: Absorption, Distribution, Metabolism, Excretion
FiO_2_: Fraction of inspired oxygen
Dd: Drug dose
Cp: Drug concentration in plasma
NAc: Nucleus accumbens
